# Artemisinin Directly Targets Malarial Mitochondria through Its Specific Mitochondrial Activation

**DOI:** 10.1371/journal.pone.0009582

**Published:** 2010-03-08

**Authors:** Juan Wang, Liying Huang, Jian Li, Qiangwang Fan, Yicheng Long, Ying Li, Bing Zhou

**Affiliations:** 1 The State Key Laboratory of Biomembrane and Membrane Biotechnology, Department of Biological Sciences and Biotechnology, Tsinghua University, Beijing, China; 2 Shanghai Institute of Materia Medica, Chinese Academy of Sciences, Shanghai, China; Charité-Universitätsmedizin Berlin, Germany

## Abstract

The biological mode of action of artemisinin, a potent antimalarial, has long been controversial. Previously we established a yeast model addressing its mechanism of action and found mitochondria the key in executing artemisinin's action. Here we present data showing that artemisinin directly acts on mitochondria and it inhibits malaria in a similar way as yeast. Specifically, artemisinin and its homologues exhibit correlated activities against malaria and yeast, with the peroxide bridge playing a key role for their inhibitory action in both organisms. In addition, we showed that artemisinins are distributed to malarial mitochondria and directly impair their functions when isolated mitochondria were tested. In efforts to explore how the action specificity of artemisinin is achieved, we found strikingly rapid and dramatic reactive oxygen species (ROS) production is induced with artemisinin in isolated yeast and malarial but not mammalian mitochondria, and ROS scavengers can ameliorate the effects of artemisinin. Deoxyartemisinin, which lacks an endoperoxide bridge, has no effect on membrane potential or ROS production in malarial mitochondria. OZ209, a distantly related antimalarial endoperoxide, also causes ROS production and depolarization in isolated malarial mitochondria. Finally, interference of mitochondrial electron transport chain (ETC) can alter the sensitivity of the parasite towards artemisinin. Addition of iron chelator desferrioxamine drastically reduces ETC activity as well as mitigates artemisinin-induced ROS production. Taken together, our results indicate that mitochondrion is an important direct target, if not the sole one, in the antimalarial action of artemisinins. We suggest that fundamental differences among mitochondria from different species delineate the action specificity of this class of drugs, and differing from many other drugs, the action specificity of artemisinins originates from their activation mechanism.

## Introduction

Malaria remains one of the major threats to human health. Nearly two billion people are at risk and likely more than one million die of the disease annually. The past years witnessed the wide emergence of drug-resistant strains in many regions, which has contributed largely to the resurgence of malaria, an ancient disease once deemed under control. Artemisinin, derived from the Chinese herb, *Artemisia annua L*, is a highly effective drug in our fight against this devastating disease [1], [2]. Its minimal clinical toxicity and fast action make it the best choice currently available for drug-resistant strains. Derivatives of artemisinin, including dihydroartemisinin, artemether, arteether and artesunate, or so called first generation artemisinin derivatives, were synthesized and have ever since been used in the treatment of malaria [3]. This class of drugs (artemisinins) contains an intramolecular peroxide bridge that is situated in the sesquiterpene lactone backbone structure and is key to the antimalarial function [4]. More recently, semisynthetic and synthetic endoperoxide analogues, some of which deviate distantly in the structural backbone from that of the original artemisinin were also made and in some cases associated with even more potent antimalarial effects. Intriguingly, despite many years of use clinically meaningful resistance to artemisinin has not yet convincingly been shown. What is more, near forty years since its discovery and with large amount of literatures published regarding its mode of action, how artemisinins inhibit the growth of malarial parasites remains an enigma and in a state of confusion [5], [6].

**Figure 1:**
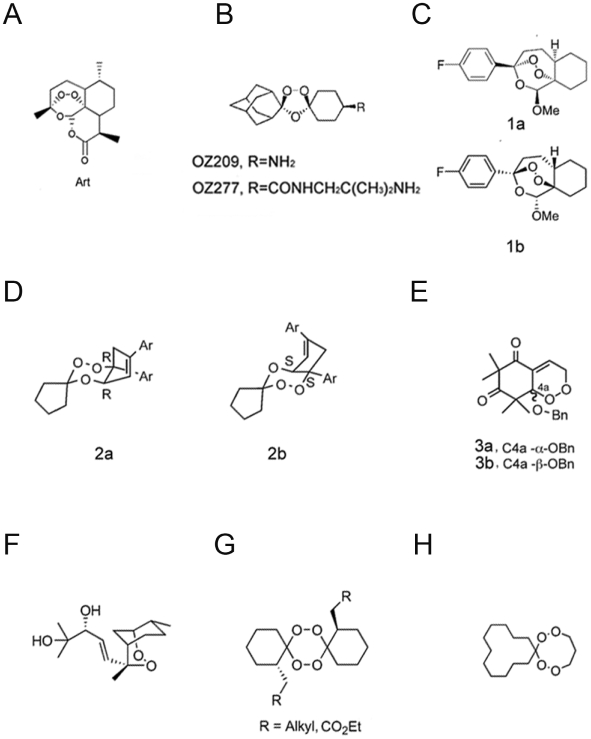
Artemisinin and its antimalarial derivatives include various peroxide compounds. (A) Artemisinin. (B) OZ209 and OZ277. (C–E) Enantiomers (1a, 1b; 2a, 2b and 3a, 3b) that have been synthesized and shown with no stereoselective difference in antimalarial activity. (F) Yingzhaosu A. (G and H) Antimalarial tetraoxanes.

The key functional part of artemisinins and their analogues is the endoperoxidic bridge, as demonstrated repeatedly by years of structural-functional research. One mystery about this class of endoperoxide drugs is that their backbones can vary dramatically without losing the antimalarial efficacy (Figure 1). On the other hand, simple change of molecular hydrophobicity at some unimportant sites can greatly diminish the drug effect [7]. More puzzling is that enantiomers of several of these compounds, including one 1,2,4-trioxanes structurally similar to artemisinin, have been made and tested against their efficacy on malaria parasites: all of them displayed no stereoselective difference in antiparasitic activity between enantiomers [6], [8], [9]. These pieces of structure-efficacy information imply that artemisinins may not achieve their functions through binding specifically to a protein-target site and consequently inhibiting its function. In other words, working models of targeting a specific protein for artemisinin's action would not fit well with what we already know about the structure-function relationship for this class of drugs. Clearly, we are facing a class of important chemicals that may work in a unique way.

To help solving these mysteries, i.e., how artemisinins work and how the excellent specificity towards malaria parasites was achieved, we previously developed a yeast (*Saccharomyces cerevisiae*) model to study the mode of action of artemisinin [10]. We found that artemisinin inhibits yeast (for convenience, yeast means *S. cerevisiae* throughout this work) growth through interfering with its mitochondrial functions. That mode of action as concluded from yeast model study, however, lacks direct biochemical supporting evidence and in particular, awaits confirmation in malarial parasites. Here, based on former clues we made in the yeast genetic studies we examined how artemisinins interact with mitochondria and how they work on malaria parasites.

## Results

### The Endoperoxide Bond of Artemisinin Is Key to Its Action in Yeast as in Malarial Parasites

**Figure 2:**
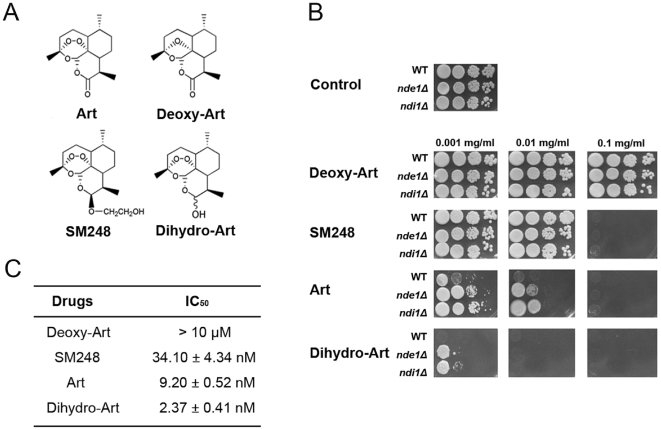
The peroxidic bridge is essential to the inhibitory activity of artemisinin in both yeast and malaria parasites. (A) Molecular structures of artemisinin and analogues (artemisinins) used in the study. Deoxyartemisinin contains only one O in the otherwise O-O bridge. (B) Inhibition of yeast growth with artemisinins. Strains of *ndi1*Δ and *nde1*Δ, with mutated internal and external NADH dehydrogenase (type II complex I) respectively, exhibit reduced sensitivity to artemisinins. (C) Inhibition of *P. falciparum* growth by artemisinins in cell culture. Data were shown as mean ± SEM.

Among artemisinin analogues with the backbone of the original artemisinin, corresponding antimalarial activities vary. In particular, deoxyartemisinin, which maintains all parts of artemisinin except the peroxide bridge, is ineffective in inhibiting malaria. We reasoned that if yeast is inhibited by artemisinin in a similar way as malaria parasites, deoxyartemisinin should not be growth inhibitory in yeast either. We thus conducted a comparative study between yeast and malarial parasites with some of these artemisinin analogues, including deoxyartemisinin, SM248, artemisinin and dihydroartemisinin (Figure 2A).

As shown in previous work and this study, deoxyartemisinin has virtually no inhibitory effect on *Plasmodium falciparum* (Figure 2C). The inhibitory efficacy of these drugs on *P. falciparum* is in the order of (from weak to strong) deoxyartemisinin, SM248, artemisinin and dihydroartemisinin. Inhibition of yeast by artemisinins can be complete and potent. To reduce growth by 50% in two days (48 hours) requires about 2–5 ng/ml of artemisinin in liquid culture [10]. However, to eliminate the growth of the normal yeast strain on plates requires much higher dosage. For example, 0.5–1 µg/ml of dihydroartemisinin is needed to completely suppress the growth of the normal yeast strain on plates (Figure 2B). This killing concentration is much higher than that in malaria parasites as we can virtually eradicate *P. falciparum* growth in cell culture with 50 folds less of the drug. It is likely that yeast mitochondrion itself is less sensitive to the action of artemisinins (by a factor of 10 as shown in isolated mitochondria experiment described below). Additionally, rapid cell growth (so more drug is needed to act quickly) and more mitochondria in yeast than malaria parasites may also contribute to this sensitivity difference.

Importantly, deoxyartemisinin has virtually no effect on yeast growth. Even when 100 times more deoxyartemisinin was used, no appreciable inhibition of yeast was observed (Figure 2B). The finding of intracellular peroxide bridge as key to the growth inhibition of both malaria and yeast suggests that mechanism of action of artemisinin in these two organisms may indeed share some key features. Worth noting is that a recent investigation reports that artemisinin's inhibition of several other protozoan parasites is weak and peroxide bond independent [11].

### Artemisinin Results Rapid Mitochondrial Depolarization in Malaria Parasites and in a Reversible Manner

**Figure 3:**
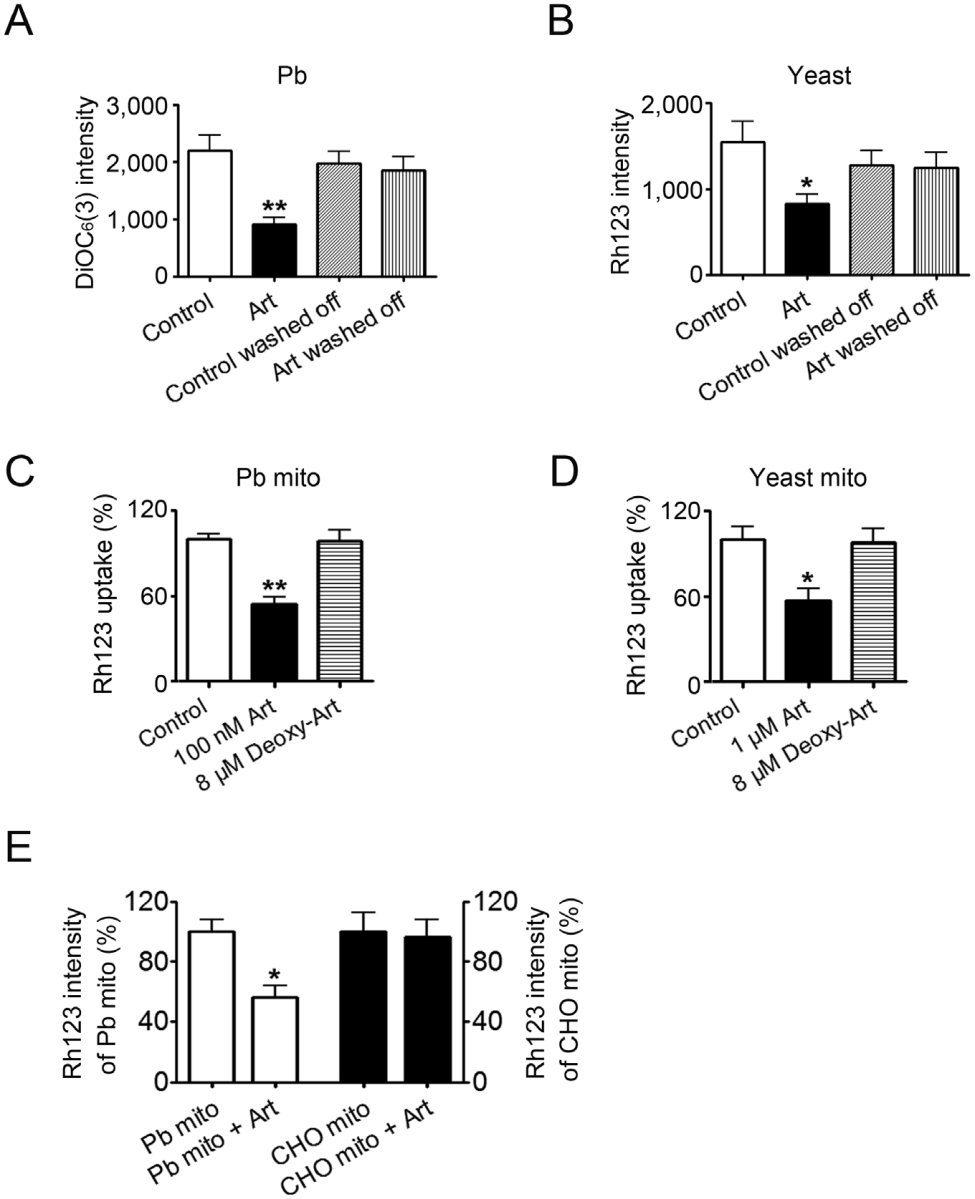
Artemisinin acts by depolarizing mitochondrial membrane of malaria parasites and yeast cells. (A,B) Artemisinin treatment resulted in loss of Δψ_m_ (mitochondrial membrane potential) in *P. berghei* (A) and yeast (B). The membrane potential could be mostly recovered by washing off artemisinin shortly after treatment. Δψ_m_ was monitored by the fluorescence intensity of DiOC_6_(3) or rhodamine 123 (Rh123). Higher fluorescence intensity of stained cells indicates higher Δψ_m_. n = 5. (C) Effect of artemisinin and deoxyartemisinin on the membrane potential of isolated malarial mitochondria. Isolated *P. berghei* mitochondria were incubated with artemisinin or deoxyartemisinin. Loss of membrane potential was apparent with 100 nM artemisinin treatment but not visible with 8 µM deoxyartemisinin. Δψ_m_ was assessed by measuring the Δψ_m_-dependent uptake of Rh123. n = 3. Art, artemisinin. Deoxy-Art, deoxyartemisinin (D) Effect of artemisinin and deoxyartemisinin on membrane potential of isolated yeast mitochondria. Isolated yeast mitochondria were incubated with 1 µM artemisinin or 8 µM deoxyartemisinin. Δψ_m_ was assessed by measuring the Δψ_m_-dependent uptake of Rh123. n = 3. Art, artemisinin. Deoxy-Art, deoxyartemisinin (E) Artemisinin selectively depolarizes malarial mitochondria but not CHO mitochondria. Mitochondria isolated from *P. berghei* and CHO cells were mixed and treated with 100 nM artemisinin. Artemisinin is not effective on the mammalian mitochondria. n = 3. Data were shown as mean ± SEM. * *p*<0.05 versus control; ** *p*<0.01 versus control.

Since artemisinin works specifically through mitochondria to achieve its inhibitory functions in yeast [10], we then asked whether this also happens in malaria. We examined the mitochondrial membrane potential of malaria parasites (*P. berghei*) treated with artemisinin. Previously reported standard scheme of treatment for malaria-infected mice is 6.2 mg/kg artemisinin injection everyday. Under this scheme, parasite cell inhibition is about 90% after 4 days [12]. We adopted this in vivo procedure to test effects of artemisinin on *P. berghei* mitochondria. Two hours after artemisinin administration, erythrocytes were isolated and *P. berghei* mitochondrial depolarization could be detected, indicating artemisinin's action on mitochondria is fast. A caveat to the interpretation of this result is that the parasites could be dying so that they lost mitochondrial membrane potential as a consequence of the death process. However, we found the loss of membrane potential was reversible at early stages: after washing off artemisinin, the mitochondrial membrane potential could mostly recover, indicating the loss of membrane potential was not secondary to cell death (Figure 3A). Similar phenomenon was observed in artemisinin-treated yeast cells (Figure 3B). These data alone suggest that under in vivo scheme, mitochondrial depolarization induced by artemisinin is an early event and not secondary to cell death (also see Discussion).

### Artemisinin Works Directly on Malarial and Yeast Mitochondria

To answer whether the depolarization of mitochondria is a direct or indirect effect, we decided to examine the effect of artemisinin on isolated mitochondria. Again we used rodent malaria parasite *P. berghei* in order to obtain sufficient amount of mitochondria. Membrane potential was monitored by rhodamine 123 uptake. As shown in Figure 3C, membrane potential of artemisinin-treated *P. berghei* mitochondria was much lower than that of the control mitochondria. Significant, yet incomplete, depolarization could be observed in the presence of as low as 100 nM artemisinin in less than half an hour. Yeast mitochondria are less sensitive to the action of artemisinin than that of malaria parasite. Depolarization could be observed in the presence of 1 µM artemisinin in less than half an hour (Figure 3D). In accordance with in vivo antimalarial and anti-yeast activity data, the peroxide bridge plays a key role for the depolarizing action because deoxyartemisinin was unable to depolarize isolated malarial and yeast mitochondria even at 8 µM (Figure 3C,D).

It has been shown that artemisinin can react with iron or heme to generate free radicals. To exclude the possibility that the acting specificity of artemisinin could be due to possible low amount of heme or other chemical contamination in the malarial mitochondria preparation, we combined together mitochondria isolated from CHO cells which stably express mitochondria-targeted DsRed and from *P. berghei*, and treated them with 100 nM artemisinin. DsRed was used as a marker to distinguish CHO mitochondria from *P. berghei* mitochondria. Mitochondrial membrane potential was monitored by rhodamine 123. As shown in Figure 3E, even when incubated together the membrane potential of CHO mitochondria was not affected while that of *P. berghei* mitochondria was decreased. Taken together, these experiments demonstrated the acting specificity of artemisinin arises from the intrinsic difference between malarial and mammalian mitochondria.

An often raised concern is about the concentration used during experiments because conceivably too much drug can cause unpredictable side effects. Inhibition concentrations, however, depend heavily on duration of treatment and extent of inhibition. To inhibit malaria growth in cell culture, depending on criteria adopted and cell strains, previous published data vary somewhat but generally about several nMs of artemisinin is required to inhibit 50% further DNA synthesis of *P. falciparum* in 24 hours after one day of prior drug incubation (for a total of two days of drug treatment time). In our hand, it took about 50 nM to inhibit more than 90% growth of *P. falciparum* within two days of drug addition based on cell proliferation (data not shown). For rodent malaria *P. berghei*, it was reported around 50 nM or slightly more artemisinin is needed for 50% growth inhibition in one day [13], [14], indicating the artemisinin concentration (100 nM) we used to observe mitochondrial effect in *P. berghei* within half an hour is physiological although less than 10 nM of the drug has been shown to inhibit 50% DNA synthesis of *P. falciparum* in 24 hours after 24 hours of artemisinin prior treatment.

### Artemisinins Are Distributed to Mitochondria

**Figure 4:**
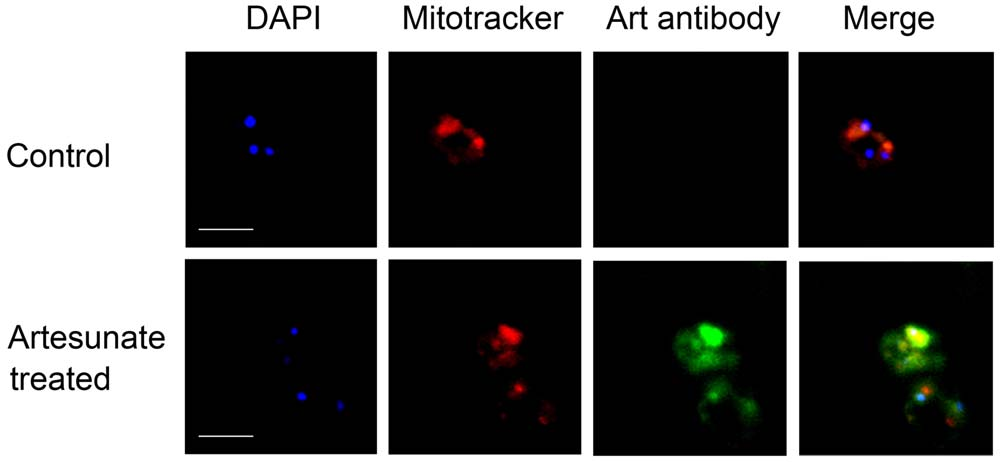
Artemisinins are distributed to the mitochondria. Immunofluorescence analysis revealed the subcellular localization of artesunate. Artesunate-treated *P. berghei* cells were stained with DAPI, Mitotracker Red and monoclonal antibody against artesuate. The top and bottom panels are cells without or with artesuate treatment, respectively. Some of the artesuate immunofluorescence signal is colocalized with mitochondria. Scale bar, 5 µm.

Although the above data indicate artemisinins can directly act on mitochondria, the next question is whether mitochondria are readily accessible to artemisinins, i.e., whether artemisinins can diffuse to mitochondria. Indirect immunofluorescence assay was utilized to answer this question. *P. berghei*-infected erythrocytes were stained with monoclonal anti-artesunate antibody [15], followed by FITC-conjugated goat anti-mouse IgG (green) secondary antibody. Figure 4 shows clearly that some of the green fluorescence signal due to artesunate staining co-localized with the red fluorescence of mitochondria. This immunofluorescence result is consistent with radioisotope observation, whereby radioactivity was seen in mitochondria as well as many other membrane structures after malaria parasites were incubated with radioactive artemisinin [16], [17], indicating artemisinins can indeed target to mitochondria and exerts their functions.

### Artemisinins Produce a Large Amount of ROS in Malarial and Yeast Mitochondria but Not in Mammalian Mitochondria

**Figure 5:**
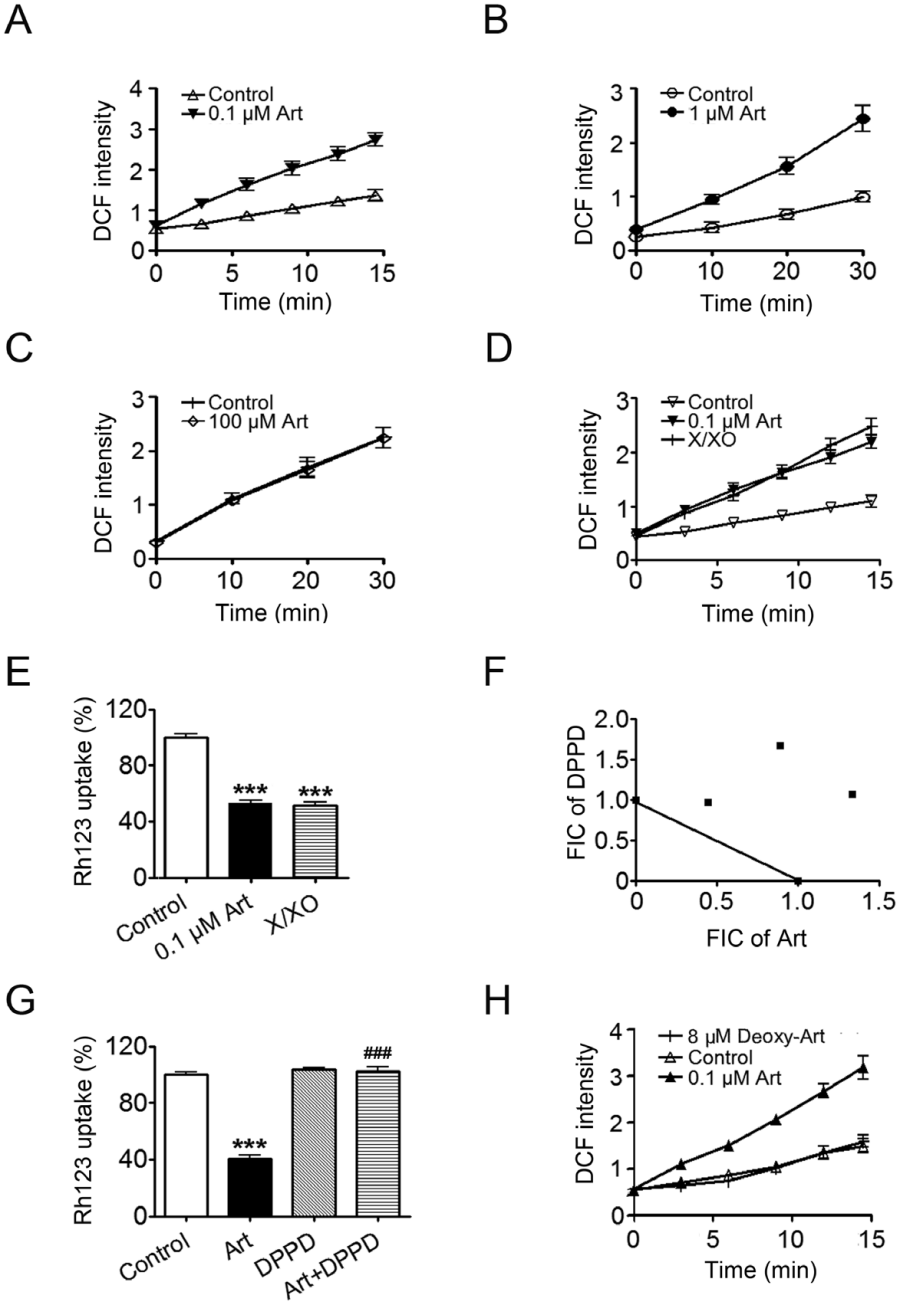
Artemisinin results in ROS production in the malaria mitochondria but not in mammalian mitochondria. (A–C) Artemisinin promotes ROS generation in isolated mitochondria from malaria parasites (A) and yeast (B) but not in mammalian mitochondria (C). ROS production was monitored by measuring DCF fluorescence intensity. (D,E) Comparable amount of ROS generated by X/XO system as artemisinin resulted in similar degree of uncoupling effect on isolated malarial mitochondria. Δψ_m_ of isolated *P. berghei* mitochondria was assessed by measuring the Δψ_m_-dependent uptake of Rh123. ROS production was monitored using dichlorofluorescin diacetate (DCFH-DA) assay. (F) An isobologram for DPI and artemisinin. Data points above the line indicate antagonism between ROS scavenger DPPD and artemisinin. FIC: fractional inhibitory concentration. (G) ROS scavenger DPPD and its effect on the depolarizing activity of artemisnin. (H) Deoxyartemisinin has no effect on ROS production in isolated malaria mitochondria. n = 3. Data were shown as mean ± SEM. *** *p*<0.0001 versus control, ^###^ *p*<0.0001 versus Art.

As a further effort to characterize how artemisinins work in detail we attempted to investigate how artemisinins depolarize mitochondrial membranes. Direct measurement of oxygen consumption of parasites or isolated malarial mitochondria revealed that 100 nM artemisnin didn't inhibit noticeably the respiratory activity, and if anything, could slightly increase it (data not shown). Thus it appears that the depolarization is mediated by contributors other than respiration interference. One likely factor is ROS. We compared the effects of artemisinin on yeast, malaria and mammalian mitochondria. Artemisinin treatment of mitochondria from malaria parasites or yeast was associated with dramatic increase of ROS production (Figure 5A,B) while importantly, it had no observable effect on mammalian mitochondria (Figure 5C) even at much higher drug concentrations. To examine whether this level of ROS would be reasonable or sufficient to disrupt mitochondrial functions, we compared it to that produced by Xanthine-xanthine oxidase (X/XO) system in which ROS was generated to induce depolarization. Xanthine oxidase converts xanthine to uric acid with the production of superoxide anion [18] and subsequently other reactive oxygen intermediates, including hydrogen peroxide, hydroxyl radicals, and singlet oxygen [19]. X/XO system has been reported to decrease the transmembrane potential of the energized inner membrane of rat myocardial mitochondria [20]. The production of reactive oxygen intermediates can be modulated by the amount of xanthine oxidase in the X/XO system. The X/XO system, when incubated with isolated *P. berghei* mitochondria, generated ROS that was comparable with artemisinin also had similar uncoupling ability with artemisinin (Figure 5D,E), indicating the ROS level associated with artemisinin treatment is sufficiently high to cause depolarization and damage mitochondrial functions. In consistent with this in vitro data, DPPD (N,N'-diphenyl-1,4-phenylenediamine), a ROS scavenger, can antagonize the antimalarial effect and the depolarizing effect of artemisinin (Figure 5F,G). Similar result was observed with another ROS scavenger edaravone (data not shown). ROS scavengers such as dithiothreitol and alpha-tocopherol have also been reported to antagonize the antimalarial effect of artemisinin [21]. Deoxyartemisinin, which lacks an endoperoxide bridge and has no antimalarial activity, had no effect on ROS production in malarial mitochondria (Figure 5H).

The fact that artemisinin produces a large amount of ROS, which is sufficient to disrupt mitochondrial functions in malarial and yeast mitochondria, together with the observation that ROS scavengers antagonize the antimalarial effect of artemisinin suggest that ROS, as produced in malaria or yeast mitochondria, mediate alone or together with other free radicals resulted from artemisinin activation to impair mitochondrial functions. This observation also indicates that the insensitivity of mammalian cells to the action of artemisinins does not arise from higher tolerability towards ROS, but inability to interact with artemisinins to generate significant amount of free radicals.

### OZ209, a Synthetic Trioxolane Structurally Related to Artemisinin, also Directly Acts on Malarial Mitochondria

**Figure 6:**
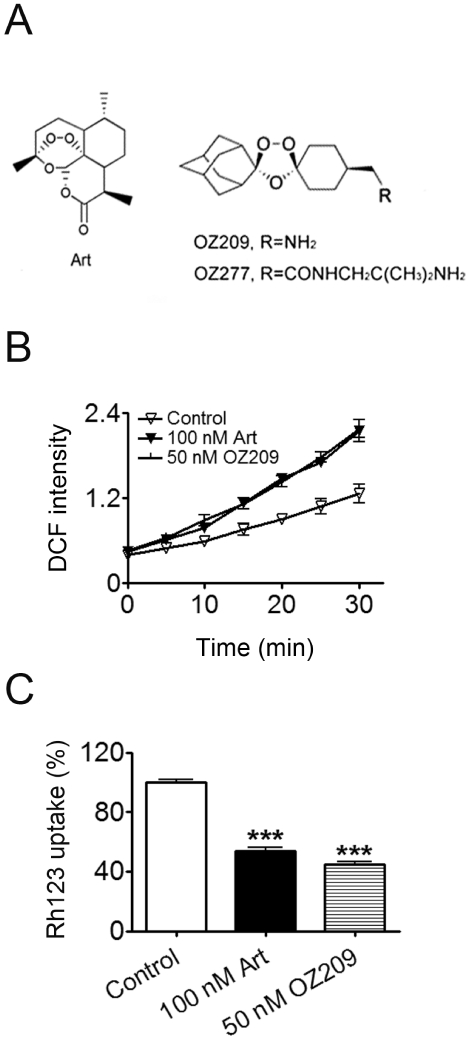
OZ209, a synthetic endoperoxide antimalarial with structurally different backbone, also affects the malarial mitochondrial functions. (A) Molecular structures of artemisinin, OZ209 and OZ277. (B) OZ209 and its effect on the ROS production in isolated malarial mitochondria. ROS production in isolated *P. berghei* mitochondria was monitored by measuring DCF fluorescence intensity. (C) OZ209 and its depolarizing effect on mitochondrial membrane. Δψ of isolated *P. berghei* mitochondria was assessed by measuring the Δψ-dependent uptake of Rh123. n = 3. Data were shown as mean ± SEM. *** *p*<0.0001 versus control.

Over the years, rich efforts have been devoted to the development of better antimalarial drug candidates based on the prototype structure of artemisinin. Notable examples include OZ277 and OZ209 (Figure 6A), two synthetic peroxide trioxolanes in which the critical peroxidic pharmacophore of the artemisinins is present within a 1,2,4-trioxolane rather than a 1,2,4-trioxane heterocycle [22]. As in artemisinins, the intracellular peroxidic bridge is also critical for the malaria inhibitory function of these synthetic peroxide trioxolanes, propelling people thinking they should share some important features in mechanism of action, and also provide an excellent test ground for the validity of working models. OZ277 and OZ209 have higher antimalarial activity than artemisinin, but are two orders of magnitude less active in inhibiting pfATP6, one previously hypothesized target protein of artemisinin [23]. As we proposed artemisinin inhibits malaria by disrupting the normal function of malarial mitochondria, we tested the idea whether the synthetic peroxide trioxolanes may also affect mitochondrial function. In isolated malarial mitochondria, OZ209 also induced dramatic ROS production. In fact, 50 nM OZ209 had similar effect to 100 nM artemisinin in increasing ROS production and depolarizing mitochondrial membrane (Figure 6B,C). The observation that synthetic peroxide trioxolane was associated with more potent ROS induction ability is consistent with its higher antimalarial activity as observed in cell culture studies [22]. This indicates that artemisinin and its related derivative OZ209 kill malaria by a similar mechanism—all through direct disruption of mitochondrial functions.

### Interference of Electron Transport Chain Affects Artemisinin's Action

**Figure 7:**
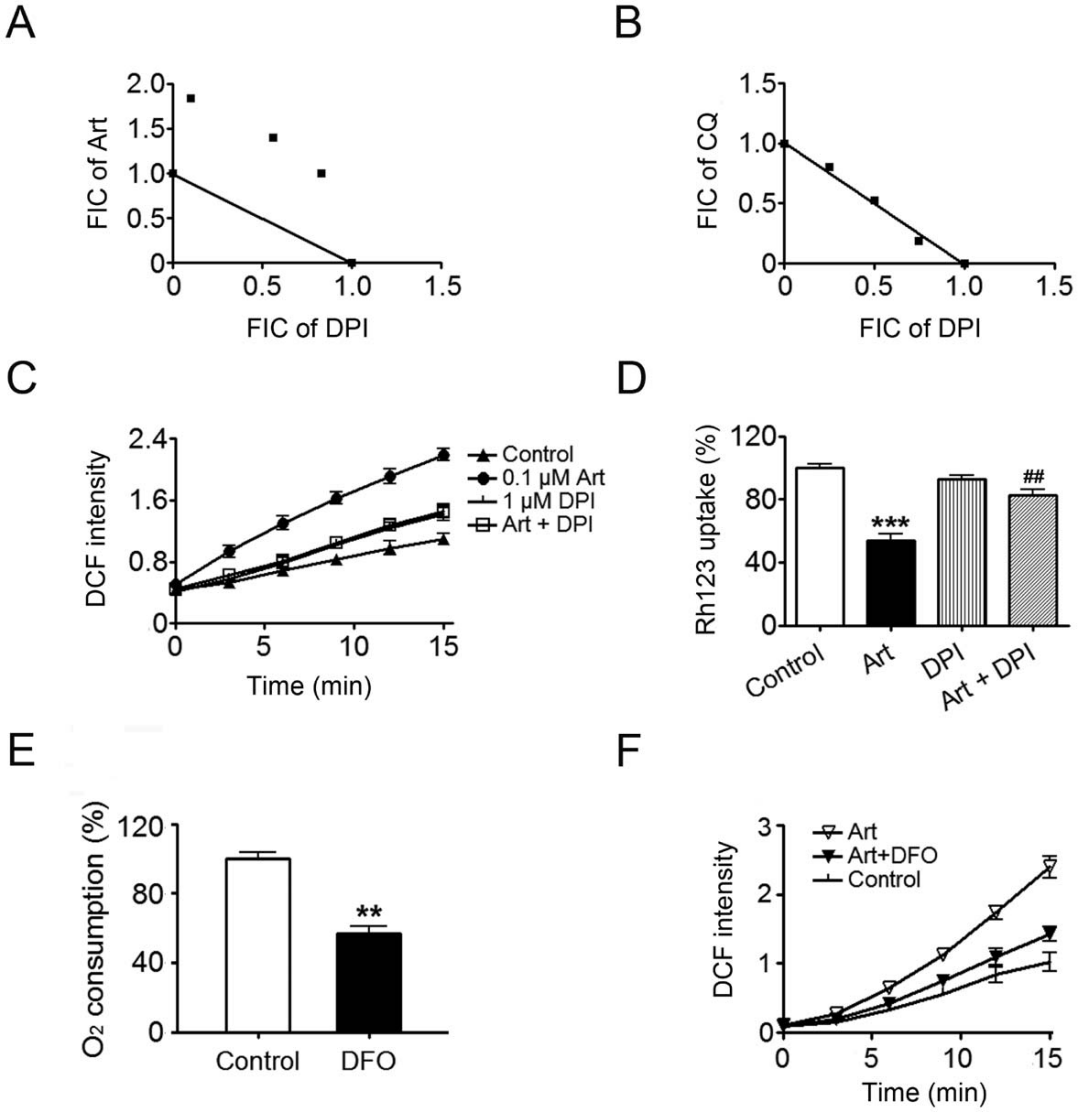
Interference of the electron transport chain affects the artemisinin's action. (A) An isobologram for DPI and artemisinin. Data points above the line indicate antagonism between DPI and artemisinin. FIC: fractional inhibitory concentration. DPI: diphenylene iodonium chloride. (B) An isobologram for DPI and chloroquine (CQ). (C) Effect of DPI on the ROS promoting ability of artemisinin. ROS was monitored using the DCFH-DA assay. (D) Effect of DPI on the depolarizing ability of artemisinin. Δψ of isolated *P. berghei* mitochondria was assessed by measuring the Δψ-dependent uptake of Rh123. (E) Iron chelator DFO inhibits ETC activity. (F) Iron chelator DFO reduces artemisinin-induced ROS production in isolated malarial mitochondria. n = 3. Data were shown as mean ± SEM. ** *p*<0.01 versus conrol; *** *p*<0.001 versus control; ^##^ *p*<0.01 versus Art.

Our prior yeast studies show deletion of genes encoding NADH dehydrogenase in ETC in yeast reduce the sensitivity to artemisinin, suggesting that ETC has some interactions with artemisinin, and probably plays an important role in the activation of artemisinin [10]. In ETC, electrons originate from electron donors such as NADH, and ultimately end in the reduction of O_2_. This electron flow can be interrupted by various inhibitors at different steps. In malaria parasites DPI has been shown to inhibit the activity of NADH dehydrogenase-an equivalent of complex I, and effectively inhibit the growth of *P. falciparum* in cell culture [24]. If the ETC in *P. falciparum* interacts with artemisinin as in yeast, we expect that DPI might antagonize the effect of artemisinin. Indeed, there was antagonism between DPI and artemisinin (Figure 7A). As a control, chloroquine, a drug that has probably no direct interaction with mitochondria, was used together with DPI. Not surprisingly, the combined effect of chloroquine and DPI was roughly additive (Figure 7B). Similar effect was also observed with IDP or flavone, two other inhibitors of NADH dehydrogenase in *P. falciparum* mitochondria (data not shown).

To obtain more direct evidence of this interaction, we then checked whether interference of NADH dehydrogenase activity affects artemisinin's effect on isolated malarial mitochondria. As shown in Figure 7C and 7D, DPI antagonized artemisinin-induced ROS production and depolarization in isolated malarial mitochondria. These data is consistent with prior yeast studies showing deletion of genes encoding NADH dehydrogenase in electron transport chain in yeast confer resistance to artemisinin and the hypothesis that ETC plays an important role in the activation and action of artemisinin.

Iron has been shown in vitro to be able to interact with artemisinin and generate free radicals. Iron chelators such as desferrioxamine (DFO) antagonize the antimalarial effect of artemisinin in cultures of *P. falciparum*, indicating iron is connected to the action of artemisinin [25]. We therefore examined how iron relates to the ROS production induced by artemisinin in isolated malarial mitochondria. As shown in Figure 7E and 7F, addition of iron chelator DFO significantly reduced ETC activity and artemisinin-induced ROS production, suggesting the key role of iron in both electron transporting and artemisinin-induced ROS production (also see Discussion).

## Discussion

Model organisms such as yeast, fly and worm have greatly facilitated our understanding of drug's action and molecular natures of many human diseases. In prior work, we established a yeast model to study the action of artemisinin. Here we present data showing although some physiological differences exist the action of artemisinin on malaria does in principle mirror many aspects of that on yeast: artemisinin can fast act to depolarize the mitochondrial membrane in malaria parasite as in yeast; rapid and dramatic reactive oxygen species (ROS) production is induced with artemisinin in isolated malarial and yeast but not mammalian mitochondria; inhibition of the NADH dehydrogenase in the mitochondrial ETC antagonizes the action of artemisinin in malaria parasite. We also show artemisinin and some homologues exhibit correlated activities against malaria and yeast, with the peroxide bridge playing a key role for their inhibitory action.

We propose in both yeast and malaria parasites, mitochondria play dual roles in the action of artemisinin: the electron transport chain stimulates artemisinin's effect, most likely by activating it, then the locally generated free radicals cause mitochondrial membrane potential loss and disrupt the normal function of mitochondria. An easily misread piece of information about our hypothesis is that artemisinin inhibits ETC. In contrast, our respiration data demonstrate that under low concentrations of artemisinin this inhibitory effect, if exists, is negligible. This means that the loss of membrane potential, a marker of affected mitochondrial functions, is not due to reduction of ETC activity. Instead, artemisinin's effect seems to be mediated by ROS or ROS together with some other free radicals as a result of artemisinin reduction. A remotely reminiscent scenario was recently reported for paraquat, which also interacts with ETC, generates ROS and inflicts damage to mitochondria [26].

Based on our hypothesis, the specificity of artemisinin is due to its selective interaction with malarial mitochondria. This interaction generates specifically in malarial mitochondria ROS, a key factor enabling the execution of the antimalarial effect of artemisinin. The peroxide bond of these molecules is essential to the generation of free radicals, and the appropriate lipophilic nature is naturally important for the drugs to cross membrane and cytosol to reach mitochondria membrane-a hydrophobic environment. Thus our hypothesis is largely consistent with the structure-function findings made through years of medicinal chemistry work that the backbones of endoperoxide drugs can vary dramatically without affecting the antimalarial efficacy.

Nevertheless, it remains unknown how exactly artemisinin is rapidly potentiated by malaria or yeast ETC but not by human or rodent ETC. Conceivably, the structuring of ETCs or properties of mitochondrial components in these organisms vary much. For example, yeast and malaria both possess similar NADH dehydrogenases which are one-polypeptide proteins and rotenone-insensitive while NADH dehydrogenases of mammalian cells are large complexes composed of many different subunits and rotenone-sensitive. The various structuring of ETCs or properties of mitochondrial components probably result in different interaction between artemisinin and mitochondria in different organisms. Because addition of iron chelator desferrioxamine drastically reduces ETC activity and correspondingly mitigates artemisinin-induced ROS production in isolated malarial mitochondria, we speculate the effect of iron and ETC might be the same thing. In other words, it is possible the iron (iron-sulfur or heme) in the ETC participates in the action of artemisinins or some subsequent steps after artemisinin activation (such as propagation of ROS). One conceivable picture, although remains to be seen, is that the ETC provides the reductive force for the possible iron-catalyzed artemisinin activation.

Morphological change in mitochondria has been found to be one of the earliest ultrastructural changes in artemisinin-treated malaria parasites [17], [27]–[29]. Notably contrast to the earlier reports, one recent publication suggested that disruption of the mitochondrial membrane potential might occur as a downstream effect of artemisinin-caused cell death as vacuolar change was discernible four hours after artemisinin treatment whereas “abnormal” rhodamine 123 marked signal was not [30]. In our hands, partial mitochondrial membrane depolarization is noticeable in about 30 minutes, significantly earlier than reported vacuolar changes, after artemisinin treatment of malaria-infected red blood cells. This rapid action, in addition to the observation of reversibility of mitochondrial membrane potential after artemisinin withdrawal, suggests that the depolarization is unlikely secondary to cell death. It is possible that the early depolarization is only partial and less radical than a more complete loss of membrane potential secondary to cell death much afterwards which causes the contradictory reports.

In summary, inspired by the initial findings we made in the yeast model we explored how artemisinin inhibits malaria parasites. Our model fundamentally differs from previous thinking that artemisinin specifically binds to a protein and inhibits it to exert its function: Artemisinin, and possibly all members of this class of antimalarials that contain the key peroxide bond, on the contrary may interact with the ETC of malaria parasites, generate local free radicals, which could impair mitochondrial functions and eventually lead to cell death. Thus, the action specificity of artemisinins is achieved through the specific mitochondrial activation in the malarial parasites or yeast, whereas the damaging action of the resultant free radicals could be local and promiscuous. It is clear that some further details of mechanism remain to be answered and refined. However, our yeast model combined with malaria studies no doubt open a new avenue in helping settling the long controversy of action mechanism of artemisinin, a mysterious antimalarial miracle.

## Materials and Methods

### Chemicals and Antibody

Artemisinin and dihydroartemisinin were from Chongqing Holley Holding Co. (Chongqing, China) and Chengdu Okay Plant & Chemical Co. (Chengdu, China), respectively. Deoxyartemisinin was prepared from artemisinin by hydrogenation. SM 248 was synthesized from dihydroartemisinin following the procedures as mentioned before [31]. Monoclonal antibody against artemisinin or artesunate was a generous gift from Dr. Baoming Wang (College of Agronomy and Biotechnology, China Agricultural University).

### Yeast Strains and Growth

Standard yeast media and growth conditions were used. BY4742 yeast strain was used as the wild type control. *nde1*Δ and *ndi1*Δ mutants in BY4742 background were from yeast deletion library (Invitrogen). For drug testing, wild type and the two mutant strains were plated on YPGE (2% glycerol and 1% ethanol as carbon source) agar plates or YPGE plates supplemented with different concentrations of artemisinin or its analogues.

### *P. falciparum* Cultivation

*P. falciparum* (isolate 3D7) was maintained *in vitro* by a modification of the method as described before [32]. The culture medium adopted was standard RPMI1640 (Invitrogen) supplemented with Albumax II (Invitrogen). Cultures were maintained in human red blood cell suspensions at a hematocrit of 5%. Parasite density was maintained below 5% parasitemia under an atmosphere of gas mixture containing 5% CO_2_, 5% O_2_, and 90% N_2_ at 37°C in a water-jacketed incubator. For the microfluorimetric method, parasites were further diluted with culture medium containing sufficient noninfected human erythrocytes to yield a final hematocrit of 2% and a parasitemia of 1%. For synchronized assay, asynchronous cultures of schizont stage parasites were pretreated with 5% sorbitol.

### Fluorimetric Susceptibility Test

Synchronized ring form cultures (hematocrit  = 2% and parasitemia  = 1%) were used to test antimalarial effects in 96-well plates. Cultures of *P. falciparum* were placed in a humidified, air-sealed container, flushed with the gas mixture as described above, and incubated at 37°C. Parasites were allowed to grow for a 48-hour incubation period. 50 µl fluorochrome mixture, which consists of SyberGreen I (Invitrogen), 10 mM Tris-HCl, 1 mM EDTA, pH 7.5 (TE buffer), and 2% Triton X-100, was then added to 100 µl cultures to liberate and label the parasitic DNA. The plates were then incubated for 20 min in the dark. The fluorescence signal was quantitated at 485 nm excitation and 538 nm emission by a microplate fluorometer (Fluoroskan Ascent, Thermo). Simultaneously, the fluorescence from negative control samples were obtained, stored and analyzed.

The effect of the interaction between antimalarial agents on the growth of *P. falciparum* culture was assessed using isobole analysis [33]. Isobologram was plotted with mean FIC to determine the interaction between agents.

### Isolation of Mitochondria from *P. berghei*

*P. berghei* was maintained *in vivo* in male mice by weekly transfer infection. Mitochondria were purified from *P. berghei* cells by differential centrifugation and Percoll gradient centrifugation [34]. Blood was collected in phosphate-buffered saline (PBS), pH 7.4, containing 10 units of heparin/ml at approximately 60% parasitemia. Red blood cells were then washed twice in PBS by centrifugation at 4°C at 1,500 g for 5 min. The parasites were freed of the host erythrocytes by incubating in PBS with 0.15% saponin and then washed at least four times. The washed parasites were resuspended in isolation buffer containing 75 mM sucrose, 225 mM mannitol, 5 mM MgCl_2_, 5 mM KH_2_PO_4_, 1 mM EGTA, proteinase inhibitor cocktail (Sigma) and 5 mM Hepes-KOH, pH 7.4. The suspended parasites were broken with glass homogenizer. To remove nuclei and large membrane fragments, the homogenate was first centrifuged at 4500 g for 5 min, and the resulting supernate was subsequently centrifuged at 24,000 g for 5 min to obtain crude mitochondrial fraction. The mitochondrial fraction was further purified by a 22% (v/v) Percoll gradient [34].

### Determination of Mitochondrial Membrane Potential

To test the effect of artemisinin on mitochondrial membrane potential of *P. berghei* cells, 6.2 mg/kg artemisinin was injected into malaria-infected mice. 2 hours later blood was collected in phosphate-buffered saline (PBS), pH 7.4, containing 10 units of heparin/ml. Red blood cells were washed twice in PBS. The mitochondrial membrane potential of *P. berghei* cells was determined by measuring the fluorescence intensity of DiOC_6_(3) using a flow cytometer (FACScan, BD). The infected erythrocytes were incubated with 2 nM final concentration of DiOC_6_(3) for 20 min at 37°C. The excitation and emission wavelengths for DiOC_6_(3) were 488 and 501 nm respectively. For each sample, 10,000 events were counted at the same flow cytometer setting. Data were shown in mean fluorescence intensity. For reversibility testing, artemisinin-treated or untreated erythrocytes were washed at least three times and then incubated in RMPI 1640 plus Albumax II for 1 hour.

The mitochondrial membrane potential of yeast cells was determined by measuring the fluorescence intensity of rhodamine 123 (Rh123). Yeast cells were incubated in YPGE with or without 16 µM artemisinin for 2 hours. At the end of incubation, yeast cells were incubated with 2 µM Rh123 for 30 min at 30°C before washing and resuspension. The mitochondrial membrane potential of yeast cells was determined using a flow cytometer (FACScan, BD). The excitation and emission wavelengths for Rh123 were 480 and 530 nm respectively. For each sample, 10,000 events were counted at the same flow cytometer setting. Data were shown in mean fluorescence intensity. For reversibility testing, artemisinin-treated or untreated yeast cells were washed at least three times and incubated in YPG for 30 min prior to the assay.

Membrane potential of the isolated *P. berghei* mitochondria was assessed by measuring the Δψ-dependent uptake of Rh123 [35], [36]. Isolated mitochondria (1 mg of protein per ml) were incubated at 30°C in a buffer containing 250 mM sucrose, 5 mM MgCl_2_, 30 mM KH_2_PO_4_, 2 mM EDTA, and 50 mM Tris, pH 7.4. Δψ was assessed by measuring the Δψ-dependent uptake of Rh123 by using a microplate fluorometer (Fluoroskan Ascent, Thermo) with excitation at 485 nm and emission at 538 nm after addition of 50 nM Rh123 to the mitochondrial suspension.

To test the specificity of the action of artemisinin on different mitochondria, mitochondria isolated from CHO cell expressing mitochondria-targeted DsRed and *P. berghei* were mixed and treated with 100 nM artemisinin. DsRed was used as a marker to distinguish CHO mitochondria and *P. berghei* mitochondria, and mitochondrial membrane potential was analyzed by rhodamine 123 intensity using a flow cytometer (FACSVantage Diva, BD).

### Determination of ROS Production in Isolated Mitochondria

ROS production in isolated mitochondria was monitored using dichlorofluorescin diacetate (DCFH-DA) assay [37]. Isolated mitochondria (1 mg of protein per ml) were incubated at 30°C in buffer containing 250 mM sucrose, 5 mM MgCl_2_, 30 mM KH_2_PO_4_, 2 mM EDTA, 2 mM α-ketoglutaric acid and 50 mM Tris, pH 7.4. DCF fluorescence intensity was measured by a microplate reader (Fluoroskan Ascent) with excitation at 485 nm and emission at 538 nm after addition of 10 µM DCFH-DA to the mitochondrial suspension. The relative units of DCF fluorescence obtained from different samples were reported as the mean±SEM.

### Oxygen Consumption

Isolated mitochondrial samples (1 mg of protein per ml) were incubated at 30°C in buffer containing 75 mM sucrose, 225 mM mannitol, 5 mM MgCl_2_, 5 mM KH_2_PO_4_, 1 mM EGTA, 0.3 mM NADH, 5 mM succinate, and 5 mM Hepes-KOH, pH 7.4. Oxygen consumption of isolated mitochondria was measured using a Clark-type oxygen electrode in a total volume of 1 ml.

### Immunofluorescence Confocal Microscopy

Artesunate-treated or -untreated infected RBCs were washed, enriched by 60% Percoll gradient, stained with 200 nM Mitotracker red (Invitrogen) and then attached to polylysine coated cover slips. The coverslips were fixed in methanol, permeabilized with 0.05% saponin and blocked overnight in blocking solution (10% heat-inactivated goat serum in PBS), followed by overnight incubation in blocking solution containing monoclonal antibody against artemisinin (dilution 1∶500). After several washes in PBS, the coverslips were incubated in blocking solution containing FITC-conjugated goat anti-rabbit IgG secondary antibody (dilution 1∶250), washed in PBS, stained with DAPI (Invitrogen) and examined by Olympus FV500 laser scanning confocal microscope.

### Statistics

Statistical differences between groups were calculated by Student's two-tailed *t* test.
